# Effect of Muscle Strength on Functionality after Shoulder Tendon Transfer in Brachial Plexus Birth Injury: Is There a Relationship between Them?

**DOI:** 10.3390/children11091125

**Published:** 2024-09-14

**Authors:** Zeynep Hoşbay, Gülsena Utku Umut, Müberra Tanrıverdi, Okyar Altaş, Atakan Aydın

**Affiliations:** 1 Department of Physiotherapy and Rehabilitation, Faculty of Health Sciences, Biruni University, Istanbul 34015, Turkey; 2 Department of Physiotherapy and Rehabilitation (English), Faculty of Health Sciences, Haliç University, Istanbul 34060, Turkey; 3 Department of Physiotherapy and Rehabilitation, Faculty of Health Sciences, Bezmialem Vakıf University, Istanbul 34093, Turkey; 4 Department of Orthopaedics and Traumatology, Başakşehir Çam and Sakura City Hospital, Istanbul 34480, Turkey; 5 Department of Plastic, Reconstructive and Aesthetics Surgery, Faculty of Medicine, İstanbul University, Istanbul 34452, Turkey

**Keywords:** brachial plexus, tendon transfer, unaffected side, muscle strength, functionality

## Abstract

Background/Objectives: Secondary problems in BPBI occur due to decreased muscle strength in the upper extremities. Comprehensive assessment methods are necessary to understand structural problems and to plan appropriate interventions in children with BPBI. We investigated the relationship between distal muscle strength, range of motion (ROM), and functionality by comparing distal muscle strength on the affected and unaffected sides in patients with BPBI who underwent shoulder tendon transfer. Methods: A total of 25 children with BPBI, 13 (52%) girls and 12 (48%) boys, aged 4–7 years (mean age: 5.98 ± 1.27 years), who had undergone shoulder tendon transfer surgery at least one year prior to the study were included. The muscle strength of the elbow, forearm, and wrist were assessed using the MicroFET^®^2 Digital Hand Dynamometer. The ROM of the elbow, forearm, and wrist were measured using the universal goniometer. The Pediatric Evaluation of Disability Inventory (PEDI) was used for functionality assessment. Results: The strength of the elbow flexor–extensor, forearm pronator–supinator, and wrist extensor muscles on the affected side was greater than on the unaffected side in all children (*p* < 0.001). No correlation was found between muscle strength, ROM, and functionality in the affected extremity (*p* > 0.005). Conclusions: Although children with BPBI have good shoulder function after shoulder tendon transfer, structural problems in the distal joints may affect their functionality during daily life. Distal joint strengthening and ROM exercises, as well as bimanual functional activities, should be included in the rehabilitation programs of children with BPBI after shoulder tendon transfer.

## 1. Introduction

Brachial plexus birth injury (BPBI) is a condition that causes primary and secondary musculoskeletal problems accompanied by flaccid paralysis of varying degrees in different parts of the upper extremity as a result of nerve damage, usually due to traction, at the roots of the brachial plexus, i.e., C5, C6, C7, C8, and T1 (and C4 and T2, if these are involved), or at any of these levels individually [1]. Paralysis can often be unilateral or bilateral and may be accompanied by secondary problems, depending on the severity of the damage. Although it is known that 80–90% of cases show spontaneous recovery in the first 3 months, recent studies indicate that functional limitations can be observed in 20–35% of them [2].

In children who do not show full recovery after the first 3 years of follow-up, changes occur in the glenohumeral joint due to muscle imbalance and soft tissue contractures. As a result of these changes, there is a limitation of movement in shoulder abduction and external rotation and losses in functions involving the movement of the glenohumeral joint, such as the movement of bringing the hand to the mouth and to the back of the neck [3]. Secondary surgical interventions such as tendon transfers and osteotomies are performed to improve these functions and increase the angle of shoulder abduction and external rotation [4]. The most frequently applied secondary surgical treatment in BPBI is the modified Hoffer technique. In this technique, the latissimus dorsi and teres major muscles are taken together and transferred to the greater tuberculum of the humerus [5,6]. Although improvements were seen in the upper extremity range of motion degrees and muscle strength of the cases after this surgical intervention, it was observed that these improvements were not reflected in the upper extremity functionality observed in these cases [7,8].

Decision making regarding the indications, timing, and treatment techniques in BPBI is a challenging process. The literature emphasizes comprehensive assessment and management of dysfunction, and it is known that the affected elbow and shoulder joints in BPBI have different functions but work together in performing activities [9]. Therefore, comprehensive assessment of the upper extremity is important for proper treatment strategies.

Although the goal of treatment is to regain control of muscle groups in the affected extremity, it is a common situation that muscles are not involved or are involved incorrectly when performing activities [10]. Strömbeck et al. [11] emphasized that the aim of both conservative management and surgical treatment is to improve the child’s functional level, but there are few studies focusing on functional rehabilitation in BPBI in the literature. Chang et al. [12] investigated the evaluation methods used in BPBI and emphasized that there may be differences between studies in terms of BPBI-specific evaluations; they suggested that future studies should also evaluate the spontaneous use of the upper extremity during daily living activities, in addition to ROM and muscle strength. In BPBI, evaluating muscle strength from an early age is important in determining the child’s functional level and possible treatment options. Muscle imbalances seen in individuals with BPBI can negatively impact functional independence and quality of life. Therefore, in our study, we aimed to investigate the relationship between distal muscle strength, ROM, and upper extremity function by comparing distal muscle strength on the affected and unaffected extremities in children with BPBI who underwent shoulder tendon transfer.

## 2. Materials and Methods

A total of 25 children with BPBI, aged 4–7 years, who had shoulder tendon transfer at least one year previously and who had not undergone any microsurgery, were included in the study. Demographic information was collected verbally from parents, and clinical data were collected from patient files; the researcher physiotherapists completed the assessments. All evaluations of the children were completed at an average of 14 months after surgery.

The study included children aged 4–7 years who were diagnosed with BPBI with unilateral C5–C6 and C5–C7 involvement who underwent tendon transfer using the modified Hoffer technique at least a year prior to the study and who were followed by a physiotherapist for at least one year, were cooperative enough to comprehend the evaluators’ instructions, and whose parents volunteered to take part in the study. The study excluded children with any neurological or orthopedic condition other than BPBI, who had undergone microsurgery related to BPBI or upper extremity surgery other than the modified Hoffer procedure, and who exhibited vision and/or hearing issues.

### 2.1. Assessments

Sociodemographic and clinical characteristics (name and surname, age, gender, dominant side, mode of birth, birth weight, and accompanying injuries) of the cases were collected using the form prepared by the researchers.

#### 2.1.1. The Mallet Scale

It is important to evaluate the global movement of the extremity and to examine functional and movement patterns in children with BPBI. The Mallet scale consists of five movements (shoulder abduction, external rotation, bringing the hand to the neck, bringing the hand to the back, and bringing the hand to the mouth); each parameter is scored 1–5. A total score is recorded for each child [13,14].

#### 2.1.2. Range of Motion (ROM)

All measurements in the study were performed by the researcher physiotherapist using a universal goniometer. Goniometric measurement is an objective method used to evaluate ROM in a clinical setting. The Kendall–McCreary criteria were followed in the measurements; each measurement was repeated three times, and their average value was recorded [15,16]. The unaffected extremity of the patient was evaluated first, and then the affected extremity was measured. All ROM measurements were performed using standard techniques.

#### 2.1.3. Muscle Strength

The upper extremity (elbow flexors and extensors, forearm supinators and pronators, and wrist extensors) muscle strength of the cases was measured using the microFET^®^2 Digital Handheld Dynamometer muscle tester (HOGGAN Scientific, LLC., Salt Lake City, UT, USA). A towel was placed between the extremity and the device to ensure full contact with the dynamometer and to prevent sensitivity. The researcher–physiotherapist applied force by asking the patient to resist as much as possible. Since the application was performed on children, the “make method” was used. In this method, the child applies force against a fixed device [17]. The application was performed on the unaffected extremity and taught to the child. The affected extremity was then evaluated. After the child was taught the method, two trials were performed on the affected extremity. After the trials, a rest break was given, and the muscle strength measurement was repeated three times. The average of the three tests was recorded in kilograms [18,19].

#### 2.1.4. Pediatric Evaluation of Disability Inventory (PEDI)

The Pediatric Evaluation of Disability Inventory (PEDI) is a clinical assessment that samples key functional capabilities and performance in children between the ages of 6 months and 7 years. It consists of three subsections functional skills: self-care, mobility, and social functions—for a total of 197 items. A total score was created by giving 1 point for the functions the subjects could perform and 0 points for the functions they could not perform [20,21].

### 2.2. Statistical Analysis

SPSS 22.0 for Windows was used for statistical analysis. Descriptive statistics were given as the minimum (min), maximum (max), mean, standard deviation (SD), and percentage (%). The Mann–Whitney U Test was used to compare the muscle strengths of the unaffected and affected extremities. The relationships between ROM, muscle strength, and functionality were analyzed using the Spearman’s Correlation Test. The level of statistical significance was accepted as *p* < 0.05.

## 3. Results

The information presented in Table 1 provides an overview of the sociodemographic and clinical characteristics of the cases. Of the 25 children examined, 13 (52%) were girls, while 12 (48%) were boys. The mean age of the patients was 5.98 years, with a standard deviation of 1.27 years. The mean birth weight was 4.25 kg, with a standard deviation of 0.53 kg. The mean height was 51.42 cm, with a standard deviation of 1.60 cm. The mean Mallet score was 17.28, with a standard deviation of 1.06. The study also found that 12 (48%) cases involved the left side, whereas 13 (52%) involved the right side. In terms of the level of involvement, 6 (24%) cases involved C5–C6, and 19 (76%) involved C5–C7.

The ROM measurements and PEDI scores of the cases are given in Table 2.

Table 3 shows a comparison of muscle strength between the unaffected and affected extremities. Significant differences were observed in favor of the affected group for elbow flexor, elbow extensor, forearm pronator, forearm supinator, and wrist extensor muscle strength (*p* ≤ 0.05).

The correlations between the ROM measurements and the muscle strength results and PEDI scores are given in Table 4. There was no correlation between the ROM measurements and the muscle strength results and PEDI scores (*p* > 0.05).

## 4. Discussion

We aimed to investigate the relationship between distal muscle strength, ROM, and upper extremity function by comparing the distal muscle strength of the affected and unaffected extremities in children with BPBI who underwent shoulder tendon transfer. As a result of the study, we found that although distal muscle strength on the affected side was greater than on the unaffected side, muscle strength was found to be neither associated with the ROM of the distal joints nor with upper extremity function.

The main causes of secondary problems in the upper extremities in BPBI are the decrease in muscle strength and sensory loss. These problems include contractures, muscle shortening, skeletal malformations, joint dislocations, limb length discrepancies, abnormal posture, and most importantly, motor coordination disorders and psychomotor developmental delays. These conditions are called developmental apraxia [22] and limit functionality in daily life, school life, and community participation [11].

Interventions ranging from physiotherapy and rehabilitation to surgery, in the light of appropriate assessment methods, are necessary to minimize structural problems and maximize the functionality of children with BPBI. In the study conducted by Pondaag and Malessy [23], an international consensus was achieved regarding valid assessment methods, including ROM measurements, muscle strength evaluations, and functionality assessments.

One of the main goals of BPBI rehabilitation is to improve functionality by minimizing strength imbalances between muscles. However, there is limited data describing the pattern or severity of muscle atrophy in the affected extremity and comparing these to those of the unaffected side [24,25]. Van der Holst et al. [24] suggest that future studies should accurately determine distal functions and participation in activities, in addition to ROM and muscle strength assessments. Therefore, we aimed to compare the muscle strength of the affected and unaffected extremities while examining the relationship between muscle strength, ROM, and functionality of the affected side in children with BPBI who underwent shoulder tendon transfer.

Muscle strength is the maximum voluntary force that a muscle group can produce and is known to be positively correlated with functional activities in children with physical disabilities. Therefore, muscle strength assessments must be valid and reliable [26]. A handheld dynamometer that can precisely monitor changes has been used to monitor muscle strength in children [27,28]. However, it has been noted that there is no protocol for using handheld dynamometers, especially in pediatric rehabilitation, and it is emphasized that the handheld dynamometer we used in our study is suitable for use in children and adolescents [29].

In a study in which muscle strength was evaluated with a dynamometer in BPBI, the muscle strength of the affected extremity of children with BPBI who underwent surgery was compared with that of the unaffected side 12 years after surgery, and it was reported that there were difficulties in the evaluation of muscle strength. It was stated that children’s cooperation, positioning, and compensatory movements were among the limitations of both manual and dynamometer measurements [30]. Similarly, in our study, although dynamometer assessment can sometimes motivate children, explaining the test to the child, preventing compensatory movements by keeping them in the appropriate position, and ensuring cooperation until the end of the test were among the difficulties we encountered.

A study by Brochard et al. [31] found significant muscle strength differences between the affected and unaffected extremities of children with BPBI. Using a hand dynamometer, the researchers measured shoulder strength and agonist/antagonist muscle balance profiles of dominant and non-dominant upper extremities. Pons et al. [32] evaluated shoulder strength in children with BPBI using a hand dynamometer but did not perform a comparative analysis between directly affected and unaffected shoulders. Similarly, this study measured elbow flexor and extensor muscles, forearm supinator and pronator muscles, and wrist extensor muscle strength and ROM in children who underwent shoulder tendon transfer. Assessment of the muscle strength of the wrist flexor was not included because wrist extensors are thought to contribute more to functionality [29].

Muscle imbalance in BPBI cannot explain the cause of paradoxical contractures, especially elbow flexion contracture, which develops when the elbow extensor function is preserved with paralysis of the flexor muscles in the elbow joint. On the other hand, decreased muscle strength in BPBI was presented as an argument against mechanical etiologies such as unbalanced or excessive muscle strength, which have been previously suggested for the pathogenesis of contracture [33]. Unlike examples in the literature, in our study, the elbow, forearm, and wrist muscle strengths in the affected extremity were greater than those in the unaffected extremity. This was thought to be due to the fact that the included children attended physiotherapy and rehabilitation programs for at least one year after surgery, and that exercises targeting the affected upper extremity were applied in these programs. Furthermore, our finding reinforced the clinical importance of a multi-dimensional strength assessment in predicting deformity and limitations in children with BPBI [31]. Future studies with a larger sample size are needed to provide more comprehensive insights.

Leblebicioğlu et al. [34] commented on the relationship between joint problems and functionality. They reported that distal joint problems may lead to an asymmetrical body image and cause difficulty in reaching an object with both hands due to the decreased length of the upper extremity. It has also been reported that forearm supination contractures may cause significant changes in body image by causing social and physical difficulties, especially in school tasks, due to keeping the hand in the supine position on the table. Pronation contractures have been reported to complicate functions such as facial and hair care. To improve these functions, distal nerve transfers have become increasingly common in BPBI, particularly in cases where shoulder function is good, but elbow flexion is inadequate. Distal ulnar and/or median fascicular nerve transfers to branches of the musculocutaneous nerve have shown favorable results for elbow flexion in the BPBI population [35]. Cases in which distal nerve transfer was performed were not included in our study, as they should be evaluated as a separate group in future studies.

To the best of our knowledge, no study has examined the effect of distal muscle strength on ROM and functionality in children undergoing shoulder tendon transfer. We believe that evaluating joint contracture formation, muscle strength, and functionality together is essential for developing an effective treatment program.

PEDI is a tool used to assess children’s ability to perform self-care activities and is effective for assessing the functionality of children with BPBI. Administration of the whole test is recommended [36]. The recovery of functions such as completing daily activities, lowering an object from above, handwriting, using a wheelchair, driving a car, and swimming depends on the recovery of elbow, forearm, or wrist functions [37]. For these reasons, we preferred PEDI for the functional assessment of children with BPBI in our study. Vaz et al. [38] reported that children with BPBI do not prefer to use the affected arm, even if their muscles are strong enough.

In our study, the distal muscle strength of the affected extremities was greater than that of the unaffected extremities. However, this difference in muscle strength was not reflected in the functionality of the children. Although the PEDI, which we used as a functional assessment tool, has been reported to be an effective assessment tool [36], it is believed that more information is needed to evaluate the quality and difficulty of movement. In addition, the lack of normative values for Turkish children and BPBI can be stated as a limitation. On the other hand, it is known that children with BPBI prefer to use their unaffected extremities when performing activities of daily living. Although the distal muscle strength of the affected extremity was higher than that of the unaffected extremity, the PEDI scores were between 158 and 184 out of 197. Some tasks in the functional skills sub-parameter could not be performed, mainly with the affected extremity. Although muscle strength and range of motion were sufficient, it was thought that the patients preferred not to use their affected extremities, and this was reflected in the functional results.

### 4.1. Clinical Applications

In our study, the distal muscle strength of the affected extremity was greater than that of the unaffected extremity. However, considering that this difference was not reflected in functional activities, it was revealed that muscle strength evaluations cannot be the most essential criterion in the follow-up of children, and multicomponent evaluations are necessary. On the other hand, it is observed that rehabilitation programs in children with BPBI focus only on improving upper extremity muscle strength and range of motion; there are fewer studies evaluating changes in activities of daily living [39]. Considering the importance of functionality in BPBI, it is essential to establish a guideline that identifies the care needs necessary to optimize upper extremity function in children with BPBI [40]. Functional assessment and treatment approaches will effectively improve the functional limitations seen in the upper extremities, as children with BPBI usually prefer to use their unaffected hands more often, “forgetting” the usable capacity of their affected upper extremities during their daily activities [38,41,42]. Our study contributes to the literature by showing that although the muscle strength of the affected extremities of children is greater than that of the unaffected side, it does not create a functional effect.

### 4.2. Limitations

The limitations of our study include the small sample size, as well as the fact that the upper extremity muscles could not be evaluated separately considering the ages of the children.

## 5. Conclusions

In our study, although children with BPBI displayed good shoulder function after shoulder tendon transfer, problems in the distal joints may affect their functionality during activities of daily living. It is important to include distal muscle strengthening, ROM exercises, and bimanual functional activities in the postoperative rehabilitation planning of children with BPBI undergoing shoulder tendon transfer.

## Figures and Tables

**Table 1:** Sociodemographic and clinical characteristics.

	Mean ± SD	Min–Max
Age (years)	5.98 ± 1.27	4–7
Birth order	1.80 ± 0.80	1–3
Birth weight (kg)	4.25 ± 0.53	3.59–5.50
Height (cm)	51.42 ± 1.60	50.0–56.0
Mallet scale scores	17.28 ± 1.06	15.0–20.0
	%	*n*
Gender (girl/boy)	52/48	13/12
Affected side (left/right)	48/52	12/13
Affected level (C5–C6/C5–C7)	24/76	6/19
Type of delivery (Doctor assisted/midwife assisted)	84/16	21/4

Min: minimum; Max: maximum; SD: standard deviation; *n*: number; kg: kilogram; cm: centimeter.

**Table 2:** ROM measurements and PEDI scores.

		Mean ± SD	Min–Max
ROM measurements (degrees)	Elbow flexion	122.80 ± 17.26	80.0–140.0
	Elbow extension	−15.80 ± 11.42	−40.0–0.0
	Forearm pronation	52.40 ± 29.47	−40.0–90.0
	Forearm supination	51.68 ± 27.75	−30.0–90.0
	Wrist flexion	64.40 ± 25.95	−30.0–90.0
	Wrist extension	46.60 ± 11.70	25.0–70.0
PEDI scores		181.68 ± 9.24	158.0–194.0

Min: minimum; Max: maximum; SD: standard deviation; ROM: range of motion; PEDI: Pediatric Evaluation of Disability Inventory.

**Table 3:** Comparison of muscle strength between unaffected and affected extremities.

		Unaffected Extremity		Affected Extremity	95% CI		*t*	*p*
	Mean ± SD	Min–Max	Mean ± SD	Min–Max	Lower	Upper		
Elbow flexor (kg)	5.48 ± 1.61	3.00–9.00	6.54 ± 1.80	3.00–10.90	−1.594	−0.545	−4.174	0.000
Elbow extensor (kg)	4.82 ± 2.16	1.70–10.10	6.71 ± 2.86	2.10–13.20	−2.541	−1.084	−5.090	0.000
Forearm pronator (kg)	4.01 ± 1.11	1.70–6.50	5.08 ± 1.31	2.30–8.60	−1.490	−0.655	−5.258	0.000
Forearm supinator (kg)	3.49 ± 1.23	1.50–5.80	4.50 ± 1.11	2.60–7.20	−1.323	−0.716	−6.875	0.000
Wrist extensor (kg)	3.76 ± 1.25	1.40–6.80	5.54 ± 1.51	2.40–8.70	−2.221	−1.272	−7.527	0.000

Min: minimum; Max: maximum; SD: standard deviation, CI: confidence interval; kg: kilogram; Mann–Whitney U test.

**Table 4:** Correlation between ROM and muscle strength and functionality.

		Range of Motion						Muscle Strength				
		Elbow		Forearm		Wrist		Elbow		Forearm		Wrist
		Flexion	Extension	Pronation	Supination	Flexion	Extension	Flexors	Extensors	Pronators	Supinators	Extensors
Muscle Strength	Elbow flexor											
	*p*	0.400	0.254	0.859	0.970	0.701	0.206	1.000	0.0	0.0	0.001	0.0
	r	0.176	0.237	−0.037	−0.008	−0.081	0.262	-	0.742 **	0.724 **	0.617 **	0.710 **
	Elbow extensor											
	*p*	0.179	0.137	0.365	0.231	0.301	0.118	0.0	1.000	0.0	0.002	0.0
	r	0.278	0.306	0.189	0.249	0.215	0.321	0.742 **	-	0.694 **	0.597 **	0.774 **
	Forearm pronator											
	*p*	0.688	0.227	0.328	0.382	0.938	0.747	0.0	0.0	1.000	0.0	0.0
	r	0.084	0.251	0.204	−0.183	−0.016	0.068	0.724 **	0.694 **	-	0.715 **	0.663 **
	Forearm supinator											
	*p*	0.448	0.395	0.981	0.631	0.828	0.672	0.001	0.002	0.0	1.000	0.0
	r	0.159	0.178	−0.005	0.101	0.046	0.089	0.617 **	0.597 **	0.715 **	-	0.819 **
	Wrist extensor											
	*p*	0.621	0.050	0.925	0.650	0.811	0.383	0.0	0.0	0.0	0.0	1.000
	r	0.104	0.396 *	−0.020	0.095	0.050	0.182	0.710 **	0.774 **	0.663 **	0.819 **	-
PEDI score	*p*	0.404	0.951	0.329	0.407	0.461	0.248	0.556	0.306	0.230	0.307	0.413
	r	0.175	−0.013	0.204	0.173	−0.154	0.240	0.124	0.213	0.249	0.213	0.171

* *p* < 0.05, ** *p* < 0.01. ROM: range of motion; PEDI: Pediatric Evaluation of Disability Inventory; Spearman’s correlation test.

## Data Availability

Due to the nature of the research and ethical concerns, supporting data are not available.
